# Exploring How People Affected by Methamphetamine Exchange Social Support Through Online Interactions on Facebook: Content Analysis

**DOI:** 10.2196/14011

**Published:** 2019-10-01

**Authors:** Daniel Ellway, Rachel Reilly, Amanda Le Couteur, James Ward

**Affiliations:** 1 School of Psychology Faculty of Health and Medical Sciences University of Adelaide Adelaide Australia; 2 Infection and Immunity Aboriginal Health South Australian Health and Medical Research Institute Adelaide Australia

**Keywords:** methamphetamine, social media, social support

## Abstract

**Background:**

Methamphetamine is an illicit and addictive psychostimulant that remains to be a significant cause of economic burden in Australia. Social media is increasingly being used by nongovernment organizations and health services to encourage the growth of social support networks among people with health-related issues. Several studies have investigated the utility of social media in providing social support to groups of people with health-related issues. However, limited research exists that explores how people who have been directly or indirectly affected by methamphetamine use social media for social support.

**Objective:**

This study aimed to determine the types of social support being sought and provided by people affected by methamphetamine when interacting with others on a Facebook page.

**Methods:**

A total of 14,777 posts were collected from a Facebook page and transferred into an Excel document for content analysis. The posts were manually coded into categories of social support using an adapted version of Cutrona and Suhr’s Social Support Behavior Code. Posts could be coded into more than one category. Saturation was reached at 2000 posts, which were used to draw inferences.

**Results:**

Emotional support was the most offered support type, with 42.05% (841/2000) of posts providing this form of support. This is followed by esteem support, which was provided in 40.40% (808/2000) of posts. Overall, 24.20% (484/2000) of posts offered informational support. Network support and tangible support were the least offered support types, with 2.25% (45/2000) and 1.70% (34/2000) of posts offering these types of support, respectively.

**Conclusions:**

This study suggests that online social support groups can be effective in challenging stigma by encouraging people affected by methamphetamine to connect with each other and talk about their struggles. This in turn represents an important step toward successful rehabilitation.

## Introduction

### Methamphetamine Use in Australia

Methamphetamine is an illicit psychostimulant that is available in 3 distinct forms: powder (speed), base methamphetamine (base), and crystalline methamphetamine (ice or crystal meth), with crystal methamphetamine being the most potent form [1]. Findings of the 2016 National Drug Strategy Household Survey showed that 1.4% of Australians aged 14 years or older reported recent use of methamphetamines [2]. Despite the overall decline in methamphetamine use between 2013 and 2016, the use of crystal methamphetamine in Australia increased from 0.4% to 0.8% between 2010 and 2016 [2]. Furthermore, the findings of the Illicit Drug Reporting System’s 2017 National Report show that crystal methamphetamine remains the most commonly used form of methamphetamine among illicit drug users in Australia [3]. The report also indicates that most participants consider all forms of methamphetamine to be easy to obtain [3]. Taken together, these findings indicate that methamphetamine use remains a prevalent issue in Australia. Research has shown that methamphetamine use is associated with higher prevalence of anxiety, major depression, and suicide than the general population [4,5]. Moreover, unlike opioids, such as heroin, methamphetamine can induce psychosis, which is characterized by auditory or visual hallucinations and persecutory delusions [5,6]. The symptoms of methamphetamine psychosis are typically brief, but severe cases can lead to hospitalization [5,7]. Chronic methamphetamine-induced psychosis is difficult to distinguish from the symptoms of schizophrenia on hospital presentation, and it can take days for clinicians to distinguish a case of methamphetamine-induced psychosis from schizophrenia [4,8]. As such, methamphetamine-related hospital presentations require prolonged admissions and substantial resources, placing a significant economic burden on the Australian health care system [8]. In addition, psychosis is sometimes accompanied by aggressive behavior [9]. Psychotic symptoms, particularly persecutory delusions, can make nonthreatening situations appear threatening to a methamphetamine user and can result in violent resolutions [9]. The difficulty in dealing with methamphetamine-related violence places a resource burden upon emergency frontline services [10]. Methamphetamine-related violence is also prevalent in the form of criminal activity [10] and domestic violence [9,11]. Thus, methamphetamine has negative impacts not only on users but also on family and the people around them.

### Social Support and Mental Health

Social support has been defined as the information that an individual receives from family, friends, peers, or strangers, which makes them feel loved, valued, or part of a wider network [12]. It has also been defined as the network of family, friends, neighbors and community members, which is available in times of need to provide psychological, physical, and financial help [13]. Previous research has examined social networks in terms of 2 main dimensions: a structural dimension, which considers network size and frequency of social interactions, and a functional dimension, which considers the emotional and practical components of a social interaction [13]. Although the quality and quantity of social networks are both important, most studies have found that the quality of relationships is a better predictor of mental health [13]. The literature indicates that social support is essential to maintaining psychological well-being. Increased social support appears to function as a protective buffer against stress from adverse life events, thereby enhancing resilience to stress [14]. Social support has also been found to be a key factor in decreased risk of depression [15,16]. Previous research found that a lack of adequate social support and smaller social networks were linked to higher levels of stress and risk of depressive symptoms [14,17].

Furthermore, 2 main explanations have been put forward to explain how social support enhances resilience to stress. The first explanation is that social support fosters healthier coping strategies and provides the individual with knowledge, improving the individual’s self-efficacy and enabling the individual to deal with stress more effectively [14,15]. The second explanation is that social support helps individuals overcome feelings of loneliness, which enables them to experience hope [14,18]. Although the exact neurobiological mechanisms of social support are not completely understood [13], it is clear from the literature that social support can positively impact mental health.

### Social Media and Online Support Groups

Social media can be defined as a group of internet-based apps that allow the creation and exchange of user-generated content [19]. The use of social media has become commonplace in Australia, with 79% of the population maintaining a social media profile [20]. Facebook is the most popular social media platform in Australia, with 95% of social media users maintaining a profile [20]. Although many people use social media to connect with family and friends, social media has also been used to form communities of specific interest [21]. Online support groups are one such example of this. With the rise of social media, an increasing number of people have turned to online support groups as an alternative to face-to-face support.

Several studies have shown that online support groups possess many advantages over face-to-face support, which explain its widespread use. Social media is accessible to anyone with an internet connection. This makes online support groups especially useful for people with limited mobility because of disability [22]. Online support groups also take place without the constraints of time, distance, and social status [22,23]. This allows for individuals to reply to messages at their own pace, which is not only convenient but also empowering for people who find face-to-face communication difficult.

Furthermore, online support groups provide individuals with safe, nonjudgmental places where they may express negative feelings that may be viewed as objectionable [16]. The option of anonymity can also facilitate greater self-disclosure because of less fear of stigmatization [15,23]. Finally, social media enables individuals to communicate with a varied range of people offering diverse perspectives and information [23]. In some cases, online support groups may be a more preferable option than family, as they are able to receive support while maintaining a comfortable emotional distance [21].

### Category Systems of Social Support

A number of studies have developed category systems to investigate the types of social support sought by individuals by using online support groups. Cobb [12] constructed a simple system that categorized social support into 3 main categories: emotional support (information signifying that one is cared for and loved), esteem support (information signifying that one is valued), and network support (information that gives one a sense of belonging). House [24] created a matrix that defined 4 broad categories of support: emotional support, appraisal support (affirmations or feedback), informational support (advice or suggestions), and instrumental support (financial or physical aid). Kalichman et al [25] constructed a similar system that categorized social support into 3 categories: emotional, informational, and instrumental support. Cutrona and Suhr’s [26] Social Support Behavior Code (SSBC) is a widely used category system that was developed to measure the frequency of supportive behaviors. The SSBC comprises 23 supportive behaviors that are encompassed by 5 broad categories of support: informational, emotional, esteem, network, and tangible support. The items were developed using previous studies of social support and were validated in a study of dyadic interaction among college students [26]. The SSBC has been adapted to studies focusing on people with disabilities [22], Huntington’s disease [23], HIV/AIDS [15], cancer [27], autism spectrum disorder [28], depression [29], and bariatric surgery patients [30].

### Methamphetamine and Social Media

Numerous studies have investigated how people use online support groups for social support. The majority of the literature indicates that informational and emotional support tend to be the most frequently offered support types, with tangible support tending to be the least frequently offered type of support [15,23,28,30]. With regard to drugs, at least one study has examined how individuals use social media to exchange information about novel psychoactive substances [31]. However, there appears to be little research focusing on how people affected by methamphetamine use online support groups for social support. Social media holds much potential for use in health care, providing people affected by methamphetamine with access to social support. The first advantage of social media is its ability to reach a specific audience regardless of location. This is especially relevant in the case of methamphetamine addiction, which is notably more prominent among Australians living in rural and remote areas. Findings of the 2016 National Drug Strategy Household Survey indicated that people in remote and very remote areas were 2.5 times as likely to use methamphetamines than those living in major cities [2]. Griffiths and Christensen’s [32] systematic review of 2 Australian Web-based mental health programs (MoodGYM and BluePages) indicated that Web-based mental health interventions are effective in reducing depressive symptoms. A study that was included in the review indicated that 20.5% of spontaneous MoodGYM users are from rural or remote areas, suggesting that Web-based interventions are relevant to people living in rural and remote areas [32]. Therefore, social media may prove useful for people living in rural and remote area, who are seeking social support for methamphetamine addiction. The second advantage of social media is its potential to challenge stigmatization, another issue that is relevant to methamphetamine addiction. Chalmers et al’s [33] study found that increases in stigmatizing media attention toward crystal methamphetamine in Australia were associated with underreporting of lifetime methamphetamine use in population surveys. Moreover, individuals affected by methamphetamine addiction in Australian Aboriginal communities often experience intense shame, which has prevented some individuals and their families from seeking help [34]. In turn, social media could provide people with access to social support without fear of stigmatization. Previous research shows that people with diseases that are considered stigmatizing, such as HIV and prostate cancer, were more likely to use online support groups for social support than people with diseases that were not stigmatizing [35]. It is reasonable to believe that people affected by methamphetamine may be willing to engage with online support groups.

Finally, research shows that social media can play an important role in changing health behavior. In Maher et al’s [36] systematic review of studies focusing on health behavior change interventions using Web-based social networks, 9 of the 10 included studies were found to have reported significant improvements in some aspect of health behavior change. This may be because of the interactive nature of social media. As social media users are required to actively generate content, social media is able to achieve higher rates of user engagement than traditional websites [36]. Taken together, these points demonstrate the potential of social media in providing people affected by methamphetamine with access to social support, which represents the first step toward rehabilitation.

### This Study

The objective of this study was to explore how people who have been directly or indirectly affected by methamphetamine use an online support group to provide social support to each other. A content-analysis approach will be used to determine the types and amounts of support exchanged by people affected by methamphetamine. On the basis of previous findings in the literature, it is hypothesized that informational support and emotional support will be the 2 most frequently offered support types and that tangible support will be the least frequently offered support type. Exploring the types of support being exchanged on the Web by people affected by methamphetamine may reveal unique insights that could be useful in developing social media resources tailored to individuals, families, and communities affected by methamphetamine.

## Methods

### Participants

The participants of this study were members who posted messages on the Facebook page *Never Give up Giving up Ice, Drugs*. This page was chosen, as it was popular at the time it was active and contained a large amount of information and experiences, which made it suitable for content analysis. The page was created by an Aboriginal Australian individual who overcame methamphetamine addiction, and it was intended to be used as a space on the Web where individuals and family members affected by methamphetamine could connect with each other. Participants were required to have a Facebook profile to post on the page and were able to request for the page administrator to post their messages anonymously, if desired. The posts themselves were available to the public and able to be read by those without a Facebook profile. Interactions on the Web took place in the form of opening posts and responding posts. The page was active from December 2014 to February 2017, and it generated a total of 14,777 posts. 5719 unique usernames were identified, which included individuals’ names, organization names, and posts labeled “Never Give up Giving up,” which were made by the administrator of the page, either for himself or on behalf of others. Owing to the Web-based nature of the data, sociodemographic characteristics of the participants were unable to be obtained. All posts were collected from the page and placed into an Excel document for content analysis.

### Ethical Considerations

Research involving social media can be an area of ethical concern because of issues of privacy, consent, and the potential for data to be misused. Researchers who have commented on these issues have acknowledged Facebook groups that require specific registration or passwords as private domains that require individual consent from participants [37]. Conversely, data from Facebook groups that do not require specific registration are considered to be in the public domain. For this study, data were collected from a Facebook page that did not require specific registration or a password. The data were available to the public, and ethics approval was therefore not required. All data have been deidentified to ensure anonymity of participants and prevent misuse of data.

### Data Analysis

Content analysis was used to determine the types and amounts of support being exchanged within the posts. Cutrona and Suhr’s [26] SSBC was adapted for use in this study. This model was chosen, as it has been used in several previous studies examining social support exchanges on social media. The 5 categories of social support and their definitions in this study were the following: informational support (ie, posts that provided advice or knowledge), emotional support (ie, encouragement or empathy), esteem support (ie, compliments or validation), network support (ie, emphasizing companionship), and tangible support (ie, offering physical or financial aid). Each category also contained a number of subcategories. A comprehensive table of all 23 subcategories and their definitions are provided in Multimedia Appendix 1. Posts were manually coded according to these categories, and these could be coded into more than one category of support. Saturation was reached at 2000 posts, at which no new information appeared to be emerging from the data.

## Results

### Overview

A total of 2000 posts were coded. Table 1 shows the frequency counts for each support category. As can be seen, emotional support was the most offered support type, with 42.05% (841/2000) of posts providing this form of support. Esteem support was also commonly offered, appearing in 40.40% (808/2000) of posts. Overall, 24.20% (484/2000) of posts offered informational support. Network support and tangible support were the least offered support types, with 2.25% (45/2000) and 1.70% (34/2000) of posts offering these types of support, respectively. In addition, 27.15% (543/2000) of posts contained information that did not fit into any of these 5 categories, and these were coded as *other*.

**Table 1:** Frequencies and percentages of posts for each support category (N=2000).

Support category and subcategory		Posts, n (%)
**Informational support**		484 (24.20)
	Advice	279 (13.95)
	Referral	29 (1.45)
	Situation appraisal	43 (2.15)
	Teaching	37 (1.85)
	Other (informational)	96 (4.80)
**Emotional support**		841 (42.05)
	Relationship	30 (1.50)
	Physical affection	109 (5.45)
	Confidentiality	0 (0.00)
	Sympathy	23 (1.15)
	Empathy	64 (3.20)
	Encouragement	360 (18.00)
	Prayer	18 (0.90)
	Other (emotional)	237 (11.85)
**Esteem support**		808 (40.40)
	Compliment	371 (18.55)
	Validation	429 (21.45)
	Relief of blame	8 (0.40)
	Other (esteem)	0 (0.00)
**Network support**		45 (2.25)
	Access	7 (0.35)
	Presence	23 (1.15)
	Companionship	15 (0.75)
	Other (network)	0 (0.00)
**Tangible support**		34 (1.70)
	Loan	0 (0.00)
	Perform direct task	2 (0.10)
	Perform indirect task	5 (0.25)
	Active participation	26 (1.30)
	Express willingness	1 (0.05)
	Other (tangible)	0 (0.00)
**Other**		543 (27.15)
	Anti-ice sentiment	40 (2.00)
	Congratulations	108 (5.40)
	Inaccessible	24 (1.20)
	Shared post	51 (2.55)
	Situation details	44 (2.20)
	Thanking	162 (8.10)
	Unrelated	114 (5.70)

### Informational Support

Informational support was separated into 5 subcategories: (1) advice, (2) referrals, (3) situation appraisals, (4) teaching, and (5) other. Advice included posts that provided guidance in dealing with challenges. A common situation that participants sought advice for in opening posts was dealing with a partner addicted to methamphetamine, which involved issues, such as domestic violence and the uncertainty of knowing whether their partners were clean (not using methamphetamine). Some examples of advice that participants provided in responding posts included the following:

Keep yourself and yr kids safe too...get legal advice and hopefully everything falls into place…good luck.

Write down all the reasons to stay with him, and all the reasons to leave him. Then make a decision.

Contact your local family violence service. You, and your children, are victims. If not for yourself, do it for them.

Buy a test on the day you think he might be on it and ask him to do it he wouldn’t refuse if he has nothing to hide best of luck.

Advice was also provided for individuals considering rehabilitation. For example, a participant expressed the importance of immediate rehabilitation in the reply below:

Rehab now. It only gets worse. Relapsing is easy, getting clean is hard. The longer the wait, the harder the work. It’s going to be hard no matter what. Anything worth having is worth the work.

Advice was also sought in opening posts for dealing with relapses. Below is a responding post to an individual who felt demotivated after experiencing a relapse of methamphetamine use:

You were and are doing so well. Relapses happen and the worst thing you can do is wallow in your mistake. I often feel the way you do and sometimes only not having the means is the only thing to stop uou so delete numbers and contacts related to it! Block n delete anyone that can/will get it for you if you ask n beg! Get tid off all paraphanalia, anything that remins you or enbles you and keep going! You can do it! Take this time to rest n self care, you’ll need it for tomorrow, it’s a new day and you’ve got work and you want to keep that going :).

Referrals included posts that directed individuals to a source of expertise. Participants often recommended websites to individuals in their replies:

We’re running a couple of sessions of our free Methamphetamine Family First Aid program later in October. The program has been developed together with affected family members. See our website for details.

Have a look at the Get Off Drugs Naturally page ‘Anonymous.’ They appear to have excellent results with a remarkably high success rate. Also read some of the success stories from those who’ve completed the program. Probably your best bet I reckon.

Participants also recommended professionals and rehabilitation programs to individuals, as seen in the replies below:

[name de-identified] & the TIMP team plus Transform Your Life! They’re wonderful people & may be near you if you are in Victoria. Best wishes,it’s tuff stuff but it's worth it!

I highly recommend Teen Challenge. I went through the Program there & have been clean for 4 years. I was a Speed Addict for 17 years. There is a Re hab called Cyrenian House in Wa and that has a Mothers Program where u can live with ur Child while u do Re hab. Praying that your daughter get the help she needs.

Situation appraisals included posts that reassessed a situation in a manner that aimed to help the recipient view the situation more positively or reveal new information that could be helpful. This was commonly offered to individuals dealing with relapse, as seen in the replies below:

It’s amazing the love & care that comes from being part of these lovely pages,hey? Relapse-just a learning curve not a life sentence...keep punching mate!!

It’s a lapse only, remember a lapse is part of recovery. Keep going and remember how far you have come already.

Teaching included posts that provided factual information. Participants offered this in reply to individuals who wanted to know how to determine whether their partners were clean:

You can tell if an addicts clean by just looking at them; there not twitching; restless; moving or anxious; there skin and eyes come back to life and they just look healthier; most addicts are easily red.

72 hours for ice rm thats min.

Urine tests are more accurate than blood tests because the drugs are excreted through the urine thus more concertrated for testing.

Other (informational) included posts that were categorized as informational support but did not fit into any of the preexisting subcategories. These posts tended to provide insights on the basis of personal experience. For example, a participant posted in a reply to another individual seeking information about rehabilitation centers:

Rehab is not a magic fix, it’s a place addicts can go to be supported through a big lifestyle change it all depends on the person if you truely want to be clean you will be, I went to rehab and have been clean almost a year, I’ve never wanted something so bad, and never been so proud of myself.

Another participant posted the following reply to an individual seeking help in dealing with a partner who had relapsed:

I only quit when I hit rock bottom and had lost contact with everyone close to me. Up until that point I would dismiss having a problem, and only promised to change- with no intentions. He has to want to quit first.

### Emotional Support

Emotional support was separated into 8 subcategories: (1) relationship, (2) physical affection, (3) confidentiality, (4) sympathy, (5) empathy, (6) encouragement, (7) prayer, and (8) other. Posts in the relationship subcategory included messages that emphasized closeness and love with the recipient and were often posted by participants to family members:

My beautiful girl it kills me 2 c u so lost like this. U need 2 fight this battle once and for all. We need our beautiful strong daughter back ur gorgous boys need ther mum back. U can fight this devil. U can get ur life back. We will always hold ur heart with us, and the best of u hasnt gone its just a little lost so please fight this devil dont let it win. We no u r trying i no it will take 1 day at a time, wher not going anywhere we will b rite here with you, for you. Luv u my daughter with al my heart & sole. Please come back 2 us xxx.

More proud than you will ever know...love you my precious son xxx.

Physical affection included posts that expressed physical contact verbally. Posts that contained hugs and kisses (“X” and “O”) were included and comprised most of the posts in this subcategory. To illustrate, a participant provided support to another individual dealing with a partner addicted to methamphetamine by posting the reply below:

Good on you for staying clean even though you are in the middle of it! That proves you have strength. now you just need to use that strength to do whats best for you and your baby. Big hugs xxx.

Confidentiality included posts that promised to keep recipients’ problems and situations in confidence. In the data, no examples of confidentiality were found. Sympathy included posts that expressed compassion or sorrow for the recipient. To illustrate, a participant provided sympathy to another participant dealing with domestic violence caused by methamphetamine addiction:

I so feel for you, makes me so so sad. You really need to stay away from him, let him hit rock bottom, because until then he will continue to hurt, lie, steal & all the rest of the heart ache that comes along from this drug, it will be hard for you, tomorrow is a new day.

Empathy included posts that expressed understanding or emphasized the similarity of the recipient’s situation to one’s own experience. The post below is a response that was provided by a participant to an individual seeking support for a partner undergoing rehabilitation while refusing to keep in contact:

I am in the same hell mate. I was kicked to the curb for much the same. My partner will never take me back sometimes things fail for more than one reason. U need to be a bit selfish mate and make yourself happy.

Encouragement was the most frequently offered subcategory (360/2000, 18.00%) of emotional support, and this included posts that were intended to instill hope and confidence in the recipient. Encouragement was offered by participants in most situations. For example, a participant encouraged an individual to persevere in looking after the individual’s children as a single parent:

Hang in there mate as long as there dad is there to love them and care for them that’s the main thing. Most the time it’s around the other way, my kids dad is the same as girlfriend. I hope she wakes up & smells the roses before its to late.

Prayer messages were occasionally offered by some participants to individuals who were undergoing difficult situations. The following is an example:

Oh you poor darlin some times your heart rules every thing...keep him at bay honey enough is enough surely you must see the warning signs you sound like a strong loving girl dont change just keep one step ahead, keeping you in my thoughts and praying this man seeks help good luck xxx.

Other (emotional) included posts that did not fit into any of the preexisting subcategories. These posts mainly included messages that expressed supportive sentiments (ie, “all the best” and “good luck”), but these also included those that expressed concern or used emojis (ie, smileys). The following is an example:

Omg your amazing...l hope your life is full to the brim with happiness thk you for sharing xx.

### Esteem Support

Esteem support was separated into 4 subcategories: (1) compliments, (2) validation, (3) relief of blame, and (4) other. Compliments represented the second-most frequently offered subcategory of all support categories (371/2000, 18.55%) and included posts that conveyed a positive assessment of the recipient’s efforts or qualities. Compliments were a common response to individuals posting about their triumphs in overcoming methamphetamine addiction:

Far out the difference you look amazing mate keep it up...I know it hard keep doing what your doing.

Good job keep it up u look fantastic now :).

Well done you...You have great strength & a winning attitude. So lovely to hear positive news. Thankyou.

Validation posts were the most frequently offered subcategory of all support categories (429/2000, 21.45%), and these included posts that expressed agreement with the recipient’s beliefs, actions, thoughts, emotions, or perspectives. As with compliments, validation was commonly offered as a response to individuals posting about overcoming methamphetamine addiction:

Good on you mate.

Proud of you--looking good--keep it up x.

Congratulations, well done, keep up every one will be proud of you.

The relief of blame subcategory included posts intended to alleviate the recipient’s feelings of guilt about a particular situation. In comparison with compliments and validation, relief of blame posts were extremely uncommon and almost exclusively offered to an individual who felt demotivated after experiencing a relapse. For example, a participant posted the following:

Don’t be to hard on yourself honey. Don’t give up. Work tmrw, be strong. It will all be good again yl see. Xxxxxx

Other (esteem) included posts that did not fit into any of the preexisting subcategories. Posts that could be coded in this subcategory were not found in the data.

### Network Support

Network support was separated into 4 subcategories: (1) access, (2) presence, (3) companionship, and (4) other. Access included posts that intended to provide recipients with means to new contacts and companions who may share similar interests or concerns. For example, a participant posted the following:

If you need some support from others in the same position as you msg me :) I run a support group for family members of ice addicts. It’s a private group and it’s a safe place to get advice :).

Presence included posts where participants offered their own presence, in the form of listening, for example, to another individual for support. This was occasionally offered by participants in various situations. Several participants offered presence in their replies to an individual who left their partner because of methamphetamine-related violence:

Im in QLD, if you feel like chatting pm me, I’m busy with kids but will msg back when I can :).

Inbox me if u need a talk love.

Good on you for turning your life around!!!:) you should be really proud of yourself. I’m really sorry though, the group I run is strictly to support family members of addicts. Feel free to msg me privately if you need someone to talk too.

Companionship included posts that emphasized the availability of other people who have similar interests or experiences. This differs from access posts, which were often written in the form of an invitation. Companionship was often offered by participants in their responses to individuals posting about overcoming methamphetamine addiction. The following is an example:

We are all here for you guys and gals.........you are absolutely wonderful - get those heads up, smile on your dial and song in your voice x.

Other (network) included posts that did not fit into any of the preexisting subcategories. Posts that could be coded in this subcategory were not found in the data.

### Tangible Support

Tangible support was support was separated into 6 subcategories: (1) loans, (2) perform direct task, (3) perform indirect task, (4) active participation, (5) express willingness, and (6) other. Loans included messages that offered to lend recipients a material object or money. Examples of loans were not found in the data. Direct and indirect task messages included posts in which participants offered to handle a task that was either directly or indirectly related to the cause of the recipient’s stress. Only 2 examples of participants offering direct assistance were found. A participant was a professional offering services free of charge to any of the other members who wanted to overcome methamphetamine addiction. The other participant was a member who offered to help an individual find support for domestic violence, which is shown in the reply below:

Where in Victoria are your family? I could find a support service that could help get you out of QLD and back to Victoria, back to support and people that can help. Even get the police to help they are really good when you need support. You’ve done the right thing I just hope you can find the strength to keep going the way you are!! much love your way xxxooo.

A total of 5 posts were categorized as indirect task messages. A total of 1 of these posts was a request from a participant to a family member to tag another family member to the page. The other 4 were requests for an individual to present as a guest speaker in a particular area:

The Riverland is in need. Are you coming this way.

Come to Perth.

Logan needs u we got nothing up here in QLD.

WA needs you too brother.

Active participation included posts that contained offers to join an individual in an activity. A majority of the posts in this subcategory were requests for other members to share websites, videos, experiences, and requests for petition signatures:

This, is a really hard thing to talk about...Please share and watch this...The feels are real, the Situation is real...ICE ADDICTION...

Please keep putting your stories out there people, this drug destroys lives! Thankyou.

I would like to submit my PETITION & put my hat in the ring to be a community member on the QLD TASK FORCE...would you PLEASE SIGN & SHARE - TIME IS TICKING - Petition for the Qld Premiere for change on how Meth (ICE) addiction is being managed in our State. The Detox/Rehabs, Mental Health Services and Judicial Systems are failing. Families of Addicts have minimal resources or support in our Qld communities. Internally Grateful <3.

Express willingness included posts that expressed readiness to help without specifying the exact nature of help that will be given. Only 1 post was found for this subcategory, which was an opening post directed to the page administrator:

Hi you are a great man person coming from the darkesnt too the light so proud of you cheers hope I can help you coming too Tasmania in any way.

Other (tangible) included posts that did not fit into any of the preexisting subcategories. Posts that could be coded in this subcategory were not found in the data.

### Other

Posts that contained information that did not fit into any of the 5 preexisting support categories in Cutrona and Suhr’s SSBC were coded as *other*. Most of these categories were not forms of support from an individual to another. Anti-ice sentiment refers to posts that expressed negative views of methamphetamine. These were not directed at any particular individual and expressed opinions that were often shared by other members of the page. Congratulatory posts were posted by participants to individuals who posted about their successes in overcoming methamphetamine addiction and remaining clean. Inaccessible refers to posts that contained links that were no longer accessible. Shared posts refers to posts in which a participant tagged another individual to direct the individual to the page. Situation details refers to posts in which a participant provided information on the participant’s situation. Thanking refers to posts where participants expressed their gratitude to another individual. Unrelated refers to posts that were not relevant to methamphetamine or the provision of social support.

## Discussion

### Principal Findings

The findings indicated that emotional and esteem support were the 2 most frequently provided support types, whereas tangible support was found to be the least frequently provided support type. As such, partial support was found for the hypothesis that informational support and emotional support would be the most frequently offered support types, as only emotional support was found to be one of the 2 most frequently offered types. The findings provided support for the hypothesis that tangible support would be the least frequently provided support type.

Informational support was noticeably less prominent in this study compared with previous studies [15,23,30]. This may be because the needs of these participants were more emotional in nature, considering the difficult and personal situations that many participants had to face. Informational support appeared to be particularly sought after by and offered to participants experiencing domestic violence and participants who relapsed into methamphetamine use. Advice may have proved especially useful to these participants in empowering them with the self-efficacy needed to handle their situations. The prominence of the posts that were categorized as informational (other) was also notable. These posts often provided insights by disclosing personal experiences. Similar findings were also noted in Evans et al’s [16] content analysis study examining postpartum depression in women. In their study, the participants tended to provide informational support in the form of personal accounts rather than refer to traditional sources of information, such as pamphlets or websites [16]. As suggested by their study, sharing personal stories may be effective, as it establishes commonalities among participants while providing information at the same time. This decreases the sense of isolation experienced [16].

The prominence of emotional support reflected previous findings in the literature [15,23,30]. This may be because of the fact that a majority of posts on the page were constructed as self-disclosure. In Wang et al’s [38] study of online social support among people with cancer, it was found that self-disclosure led to the perception of emotional needs, which elicited emotional support-type responses. In contrast, asking questions led to the perception of informational needs, eliciting informational support-type responses [38]. However, it must be noted that the 3 most prevalent subcategories found in this study were encouragement, emotional (other), and physical affection. Encouragement was particularly salient, and this is likely because of the nature of the page, which was set up for people affected by methamphetamine to connect with and support each other in their efforts to overcome addiction. Emotional (other) was largely represented by messages, such as “all the best,” and physical affection was similarly expressed through *X* ’s and *O* ’s. It is more likely that the prominence of emotional support is explained by how ubiquitous these expressions were. Nonetheless, these subcategories were important, as they appeared to play an important role in building close relationships on the Web.

The prominence of esteem support was a finding that appeared to be unique to this study. Esteem support was predominantly offered in the form of validation or compliments, both of which were found to be the most frequently offered subcategories of all 22 types established in Cutrona and Suhr’s SSBC. It may be that that esteem support was salient in this study, as people affected by methamphetamine are unable to find this type of support easily in their everyday lives because of the stigma of methamphetamine [34]. As Beck et al [29] suggested in their study of online support groups, the increased need to exchange emotional support could be driven by efforts to challenge stigma. Similarly, the prominence of esteem support in this study could be the result of the participants’ efforts to challenge stigma through acknowledging and celebrating each other’s achievements and personal victories in overcoming methamphetamine addiction.

In comparison with the 3 support categories discussed above, network support and tangible support represented an extremely small amount of the data. Although the lower prominence of network support was expected, it was surprising to find that network support was significantly less prominent than esteem support. This finding is inconsistent with the literature [15,23,28]. A potential explanation for this is that the participants’ needs for network support were already addressed by being active on the page. Coulson et al [23] also noted in their study that network support became less salient over time and suggested that this could have been because of the fact that participants’ network support needs were met simply by participating in the online support group. The infrequent provision of tangible support was consistent with the literature, and this may be explained by the unfeasibility of providing such support on the Web, particularly in the case of methamphetamine addiction. As noted by Atwood et al [30], this may also reflect the unsuitability of the tangible support category, as developed by Cutrona and Suhr, to online support groups.

### Implications

The findings of this study demonstrate that online social support groups represent viable opportunities for people affected by methamphetamine to find support and provide insight into the types of support exchanged on the Web by these individuals. Esteem and emotional support appear to be the most relevant and valued types of support, and informational support was also an important function of the page. This knowledge could help inform Australian health care providers in developing Web-based resources tailored toward individuals, families, or communities struggling with methamphetamine addiction. A key implication of the findings is that these resources can be effective in challenging the stigma of methamphetamine use by encouraging people to connect with each other and talk about their struggles. Provided with a platform where they can offer each other esteem and emotional support and share personal experiences, people affected by methamphetamine can reduce their feelings of isolation and experience hope. These benefits help individuals to deal with stress, and they are also critical factors that represent the first step toward rehabilitation [34].

### Strengths and Limitations

A strength of this study was the sample size. Even though a subset of 2000 posts were used out of the total 14,777 posts, the subset was nonetheless large in quantity and resembled sample sizes used in other content analysis studies examining online social support [23,30]. A second strength of the study was the use of a validated theoretical framework of social support with well-defined categories. This ensured that posts were consistently categorized and that results were reliable. However, there are a number of potential limitations in this study, which must be taken into consideration. First, the data were coded manually, without the use of a text-mining tool. Though saturation was claimed at 2000 posts, the use of a text-mining tool might have revealed information from the data that was possibly overlooked during the manual coding process. Second, the data were not always neatly categorizable. The amount of detail in these posts meant that many provided more than one type of support. As such, a majority of posts could not simply be coded into 1 category, which was done in previous studies, using Cutrona and Suhr’s SSBC framework. Third, interrater reliability was unable to be obtained for the data. This would have considerably increased the reliability of the results. Finally, the nature of the data meant that the author had to make active judgements in deciding which support categories’ posts were coded into. It is inevitable that the author’s personal interpretation of posts will have shaped some coding choices. Nonetheless, all efforts were made to ensure objectivity by referring to the SSBC framework.

### Conclusions

This study represents one of the first few studies to examine how people affected by methamphetamine interact with online social support groups. The findings show that people affected by methamphetamine particularly valued and benefitted from esteem and emotional support, as well as informational support. The next step for future research is to determine whether these findings are generalizable by replicating the study, ideally with the use of a text-mining tool and greater attention paid toward interrater reliability. Future research should also explore these interactions on the Web in greater depth, using a thematic analysis. This may reveal further insight that may be useful for Australian health care providers in developing Web-based resources to address families and communities affected by methamphetamine.

## Appendix

Multimedia Appendix 1 Definitions of social support typology classifications.

## Abbreviations

SSBC: Social Support Behavior Code
