# Supplementary material for: Exploring How People Affected by Methamphetamine Exchange Social Support Through Online Interactions on Facebook: Content Analysis
Source: JMIR Ment Health. 2019 Oct 1;6(10):e14011. doi: 10.2196/14011 (PMC6774572; doi:10.2196/14011)
Supplement: Multimedia Appendix 1 [file mental_v6i10e14011_app1.docx]

| *Support Type* | *Definition* | |
| --- | --- | --- |
| *Informational Support* |  |  |
|  | Advice | Messages that suggest courses of action or guidance for coping with methamphetamine challenges and other difficulties. |
|  | Referral | Messages that provide a source of expertise or information. |
|  | Situation Appraisal | Messages that help to reassess or redefine circumstances, often in a manner that helps make them more positive or reveal new information that could be helpful. |
|  | Teaching | Messages that provide factual information about the challenge the recipient is facing, or about the skills needed to deal with the situation. |
|  | Other |  |
| *Emotional Support* |  |  |
|  | Relationship | Messages that emphasise closeness and love in the relationship with the message recipient. |
|  | Physical Affection | Messages that express physical contact verbally. |
|  | Confidentiality | Messages that promise to keep the recipient’s problem in confidence. |
|  | Sympathy | Messages that express compassion or sorrow for the recipient. |
|  | Empathy | Messages that express understanding or emphasise the similarity of one’s own experiences to that of the recipient. |
|  | Encouragement | Messages meant to provide the recipient with hope and confidence. |
|  | Prayer | Messages that offer to pray for someone who is suffering or needs help. |
|  | Other |  |
| *Esteem Support* |  |  |
|  | Compliment | Messages that convey a positive assessment of the recipient and/or his or her abilities. |
|  | Validation | Messages that express agreement with the recipient’s beliefs, actions, thoughts, emotions or perspective on a situation and messages that acknowledge agreement or common ground with the message sender. |
|  | Relief of Blame | Messages that aim to alleviate another’s feelings of guilt or absolve them of responsibility for a situation. |
|  | Other |  |
| *Network Support* |  |  |
|  | Access | Messages that provide the recipient with access to new contacts and companions, usually because they have similar interests or concerns. |
|  | Presence | Messages that offer to “be there” for the recipient, for example by listening to them or spending time with them. |
|  | Companionship | Messages that emphasize the availability of companions who have similar interests or experiences. |
|  | Other |  |
| *Tangible Assistance* |  |  |
|  | Loan | Messages that offer to lend a material object or money to the recipient. |
|  | Perform Direct Task | Messages that offer to perform a task directly related to a stressor. |
|  | Perform Indirect Task | Messages that offer to take over a task not directly related to the stressor, but which will help the recipient deal with the stressor. |
|  | Active Participation | Messages that offer to join the recipient in an activity. |
|  | Express Willingness | Messages that express readiness to help, without specifying the exact nature of assistance that will be given. |
|  | Other |  |
